# Understanding large language models demands distinguishing human projection from machine cognition

**DOI:** 10.1038/s44271-026-00508-6

**Published:** 2026-07-16

**Authors:** Lingyu Li, Yan Teng, Yingchun Wang, Xia Hu

**Affiliations:** Shanghai Artificial Intelligence Laboratory, Shanghai, China

**Keywords:** Philosophy, Human behaviour

## Abstract

Current efforts to understand Large Language Models (LLMs) are largely metaphorical. Researchers map LLMs onto familiar domains, from physics and neuroscience to psychology and sociology, each illuminating specific facets while obscuring others. We chart these metaphors across mechanistic, behavioral, and interactive scales and delineate their explanatory boundaries. Crucially, this metaphorical projection creates a recursive loop of anthropomorphism, fueling the “genuine understanding” versus “pattern matching” impasse. As an alternative approach, we propose machine experientialism, positing that LLMs build their own form of understanding from training corpora. The priority shifts from cataloging LLMs’ human-like traits to uncovering their distinct logic that emerges from this text-based world.

## Abstract (web summary)

This Perspective proposes machine experientialism, positing that LLMs build their own form of understanding from training corpora.

## Introduction

We are dealing with a strange new entity. It can win a gold medal in the International Mathematical Olympiad^1^, yet struggled until recently to count the number of ‘r’s in ‘strawberry’. It is capable of resolving complex GitHub issues^2^, but can easily fail at simple logical puzzles^3^. Mechanistically, it is an autoregressive next-token predictor, yet internal probing suggests complex reasoning and emergent introspection during generation^4,5^. Although it lacks human embodiment and social history^6^, countless users are genuinely falling in love with it. As the Large Language Model (LLM) is increasingly deployed in diverse domains, from everyday applications to frontier scientific research, the question becomes more complicated: what on earth is an LLM?

Studies attempting to answer this question exhibit a compelling interdisciplinary trend. For instance, psychology provides scales and tests to evaluate LLM’s cognitive biases^7^; neuroscience investigates the features and circuits within LLM^8^; statistical physics provides a complexity description of the emergent ability^9^ and scaling law^10^; and economics likens LLM to a market that aggregates dispersed knowledge, where the ‘token’ serves as both a syntactic unit and a form of price signal^11^. These inquiries are fundamentally metaphorical: by mapping the target domain of LLM onto a source domain from the established discipline, researchers import that domain’s concepts, measurements, and explanatory schemas that bring specific aspects of LLM into sharp focus. From a cognitive linguistic perspective, grasping novel concepts by grounding them in more familiar experiences is a natural cognitive process of humans – what Lakoff and Johnson propose as ‘metaphors we live by’^12^.

However, this metaphorical strategy inherently conceals a twofold challenge. First, as each metaphor foregrounds a selective slice of LLM, it inevitably backgrounds others. Second, metaphors are systematic by nature. In borrowing a single concept, we often unconsciously inherit its family of related ideas, including those potentially misleading for the new context. For example, the metaphor ‘love is a war’ captures the strategic dynamics in a relationship but also obscures its cooperative dimensions and can even position the partner as an opponent. These two facets manifest in our current predicament with LLM. On one hand, the slice-by-slice focus leads to a fragmented landscape of understanding, where insights from different disciplines are difficult to reconcile into a coherent picture. For instance, a mechanistic ‘physics of LLM’ cannot predict the model’s impact on social equality. On the other hand, hidden yet unverified assumptions proliferate. The most prominent example is anthropomorphism: the metaphor of LLM as a human mind. By anthropomorphizing the artifact trained on human expressions and fine-tuned to mirror human traits, the distinction between LLM’s intrinsic capability and our cognitive projection becomes blurry, sparking long-lasting debate in the field.

**Fig. 1:**
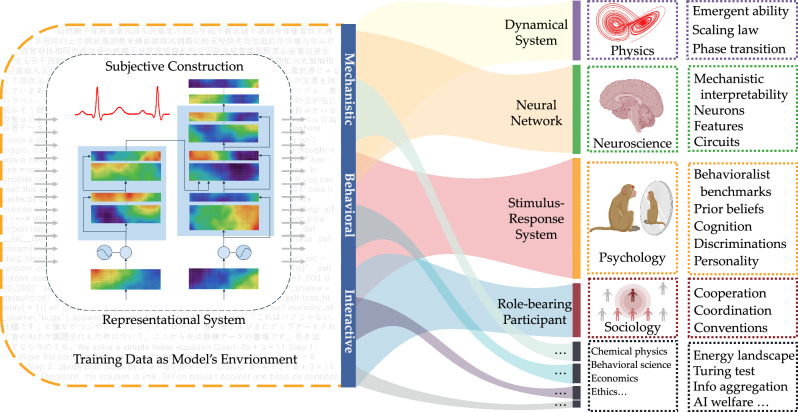
Overview of machine experientialism. From the view of machine experientialism, LLM constructs its internal reality from data through its representational system. Different disciplinary lenses metaphorically capture specific slices of this model at mechanistic, behavioral, and interactive scales. We partially created this figure using BioRender (BioRender.com).

Since each lens illuminates specific aspects of LLM while ignoring others, we argue that the solution is to understand why a particular metaphor works and where its explanatory power ceases. By distinguishing what it is from what we perceive it to be like, we can harness their explanatory power without inheriting unnecessary assumptions and collectively access a holistic understanding of LLM. To achieve this, we chart the current landscape of interdisciplinary metaphors, including physics, neuroscience, psychology, and sociology, organizing them across mechanistic, behavioral, and interactive scales, as illustrated by Fig. 1. We aim to identify the explanatory contribution and theoretical boundary of each, thereby providing an integrated understanding of the LLMs.

Regarding the recursive loop of anthropomorphism, we then extend the concept of experientialism to the non-human domain, a stance we term machine experientialism. Experientialism here will be shorthand for Lakoff and Johnson’s conceptual framework: a cognitive philosophy drawn from their theory of metaphors^12^. Experientialism holds that understanding emerges from how we interact with environment through our bodies. Machine experientialism frames LLM’s cognition as the construction of understanding from its textual environment through its Transformer architecture. This reading helps move beyond the current ‘genuine intelligence vs. pattern matching’ dichotomy and reveals the underlying category errors of over-anthropomorphism. Ultimately, this view calls for shifting the stance from how closely the LLM approximates the human mind to how its unique internal reality is constructed.

## Interdisciplinary metaphors as basic cognitive tools for understanding LLMs

Articulated by George Lakoff and Mark Johnson in *Metaphors We Live By*^12^, metaphor is not merely a figure of speech but also a cognitive tool governing how we think and act. They argue that our conceptual system is largely metaphorical in nature, a cognitive process through which we understand one unfamiliar conceptual domain in terms of another familiar domain. The conceptual metaphors are systematic cross-domain mappings. For instance, the saying ‘time is money’ leads us to believe we can waste, save, invest, allocate, or run out of this abstract physical metric. They are not arbitrarily established but grounded in our basic experience. The ‘more is up’, for instance, likely originates from the physical experience of stacking objects: the physical level rises as the quantity increases. Applying this physical logic, we say the price/mood/volume is going ‘up’. This cognitive process advances the philosophy of *experientialism*, a middle path between objectivism and subjectivism, claiming that understanding is not simply a given property of the world waiting to be discovered, nor an arbitrary creation of an unconstrained mind. Instead, it emerges from the interaction between the two. That is, the same external reality mediated through different representational systems gives rise to different subjective understandings.

Reflecting on the LLM field, when speaking of ‘attention mechanism’, ‘token’, ‘training’, ‘hallucination’, or ‘agent’, we are using such metaphorical expressions. The answer to ‘what on earth is an LLM’ is shaped and constrained by the metaphors we use to model and thus interpret it. Researchers from diverse backgrounds proactively adapt concepts and tools from their established disciplines to analyzing LLM, including but not limited to neuroscience (brain)^5^, chemical physics (molecular system)^13^, statistical physics (complex system)^9^, information theory (informational channel)^14^, cognitive psychology (epistemic subject)^15^, behavioral science (interactive agent)^16^, economics (market)^11^, and sociology (sociotechnical system)^17^. The choice of source domain provides the conceptual scaffolding, determining the questions that can be asked, the phenomena that are foregrounded, and the insights that can be adequately accessed. This vibrant intellectual borrowing, while fruitful, has also created a fragmented landscape. It fostered a condition where different metaphorical camps can often talk past each other. These metaphors fueled an explosion of publications but also sowed division within the research community. Paradoxically, the very efforts aimed at interpreting LLM seem to instead muddle a holistic understanding.

This is where the theoretical tools of metaphor analysis can help us clear the confusion. Instead of viewing them as competing opinions, we can harmonize them by mapping out which metaphor highlights which slice of the LLM. Different observers encounter LLM from different observer experiences and at different scales across computational architecture, task executor, and interactive agents. Although the choice of analytical scale depends on the observer’s perspective and research interests, we can heuristically organize current understanding of LLM at mechanistic, behavioral, and interactive scales. Notably, identifying these studies as metaphorical does not imply that the research conducted within them lacks formal rigor. Table 1 outlines four representative disciplinary metaphors we analyze in the following sections.

**Table 1:** Representative metaphors grounded in technical realities: minimal assumptions and unverified boundaries

Metaphor	Technical focus	Minimal assumption	Unverified extension
Physics/Dynamical systems (Mechanistic)		*LLM is a*	
	Dynamics of the whole parameter set during training or generation	High-dimensional system with myriad coupled units	Passive physical object, devoid of understanding or normativity
Mechanistic interpretability inspired by neuroscience (Mechanistic)	Causal mechanisms by which internal components implement specific tasks	Information-processing network with internal parts mapped to functional roles	Artificial brain sharing human affective, embodied, and homeostatic constraints
Behavioral/Cognitive psychology (Behavioral)	Input-output correlation between the prompt and response	Stimulus-response system with certain internal representations that can be probed under controlled conditions	Human-like mind with consistent personality traits, emotions, or self-identity
Sociology/Multi-agent interaction (Interactive)	Agents in real-world applications where LLM serves as cognitive core in autonomous loop	Role-bearing participant in interactive or communicative settings	Social subject with stable commitments, inherent interests, and legible intentions

## The mechanistic view from within

Mechanistically, LLM primarily refers to the Transformer architecture^18^. The core idea is the self-attention mechanism which, for every word in a sentence, computes how strongly the word relates to other words, allowing LLM to track relationships across long stretches of text and generate fluent human language. The architecture arises from *pre-training*, i.e. autoregressive language modeling (i.e. predicting the next token) from massive corpora^19^, and *post-training* to align its outputs with specific preferences or guidelines via supervised fine tuning, reinforcement learning, or test time scaling^20^. However, such a specification provides little mechanistic understanding of LLM as a complex engineering artifact, with billions to trillions of parameters, tens to hundreds of layers, millions of training steps, heterogeneous training data, and continuous versioning. Researchers thus turned to other scientific domains that have long histories of dealing with such similar complex systems, especially physics and neuroscience.

### Physics of LLM

This view adopts a macroscopic viewpoint, studying how LLM evolves as a whole during training or generation and seeking general principles governing it. The training process is seen as navigating a high-dimensional spin-glass energy landscape and most local minima are effectively equivalent to global minimum, which explains why large networks could generalize well^21^. During training, deeper layers form higher-level abstractions, described by a renormalization group flow where the network iteratively coarse-grains features^22^, or a compression phase as optimizing an information bottleneck where model discards irrelevant input details to achieve generalization^23^. Model’s reasoning process during generation can be modeled with stochastic differential equation as drift-diffusion between metastable phases, allowing for predicting misaligned states.^24^. The physics perspective provides a top-down understanding of model’s internal dynamics and emergent properties. But it offers less resolution when the focus is on specific implementations of concrete concepts and tasks.

### Neuroscience of LLM

Moving from a statistical whole to functional parts, Neuroscience of LLM presents a microscopic perspective, also known as Mechanistic Interpretability. It treats a trained model as a brain to be dissected, aiming to detect and steer neurons, features, and circuits corresponding to specific behaviors. Akin to ‘grandmother cell’ intuition in neuroscience, researchers initially focus on neurons or attention heads for linguistic features, such as ‘knowledge neuron’^25^, ‘safety neuron’^26^, and ‘multilingual neuron’^27^. Exactly as the challenge to the ‘grandmother cell’ hypothesis, this approach encounters the problem of poly-semanticity, where a neuron activates for multiple concepts^28^. Linear probes provide a supervised tool to decode how target attributes are represented, e.g. space and time^29^, by training simple linear classifiers on layer-wise hidden states^30,31^. Sparse decomposition techniques offer an unsupervised way to find mono-semantic features with sparse autoencoder and dictionary learning^32^. Circuits are constructed to explain how models perform specific tasks, which are subgraphs where nodes are interpretable features and edges are their causal interactions. Attribution graphs visualize these circuits for individual prompts by tracing linear feature interactions^8^, uncovering sophisticated mechanisms such as multi-hop reasoning, planning, and post-hoc rationalization underlying LLM at the mechanistic scale^5^. This bottom-up perspective maps functions of interest to mechanistic components. But it can be too specific to the model instance and tested prompts, thus facing challenges including poor transferability across model families, computational explosion of large-scale models, and re-introduction of the complexity of data.

Yet, these mechanistic viewpoints cannot fully grasp model’s behavioral patterns in real-world application. For instance, to inspect an LLM’s reasoning ability, a well-designed benchmark is far more direct, scalable, and practical than purely mechanistic analysis. Also, as frontier models become increasingly proprietary, mechanistic approaches may be constrained, channeling research toward behavioral and interactive scales, which risks shaping the types of LLM understanding of general researchers and public.

## The behavioral view from without

Behaviorally, LLM is a general request responder where the model’s behavior is its generative process, which is largely shaped at runtime by the context. The user’s input, comprising instructions, few-shot examples, and system-level rules, sets a specific role (e.g. a helpful assistant), task (e.g. solve a math question), and desired behavior (e.g. think step by step)^33^. LLM then begins autoregressively predicting the probability distribution of the next token, and a specific word is selected according to current decoding strategy and sampling strategies until the end-of-sequence token. To make the generation more responsive, engineering optimizations include quantization, speculative decoding, and parallelism, further introducing uncertainty of model’s behavior^34^. At this scale, LLM transitions from a technological architecture to a functional product used by billions of users. Consequently, on the one hand, understanding its real-world performances from mechanistic scale becomes impractical, giving rise to an explosive growth of benchmarks in the field. On the other hand, a technology achieves maturity only when its internal complexities are black-boxed, allowing users to interact solely with its input and output^35^. Billions of parameters hidden from view, the behavior-level, language-mediated, input-output entity invites a cognitive-psychology metaphor to understanding it. Specifically, as summarized in Table 1, cognitive psychology of LLM treats LLM as an agent whose input-output correlation between prompts (stimulus) and generations (responses) can be probed under controlled conditions to infer the agent’s internal representations.

By adapting well-established experimental paradigms, this approach charts LLM’s representational and processing properties from observable behaviors^36^. For instance, Markov Chain Monte Carlo with people paradigm^37^ recovers prior concept representations within LLMs^38,39^; similarity judgment task suggests that LLMs can predict human perceptual similarity judgements across multiple modalities (e.g., auditory and visual)^40^; a two-step-reversal bandit task indicates LLM’s weakness in reflective belief updating^15^; deliberation-harmful tasks reveal mixed performance effects of chain-of-thought (CoT)^41^; judgments of learning reveal the divergence between humans and LLMs in metacognitive monitoring^42^; decision-making tasks indicate an additive bias in LLMs^42^; prototype theory inspired study reveals distinct conceptual representations within LLMs^43^. By grounding comparison in measurable behaviors, these experimental methods can chart both the convergence and divergence between human and machine cognition without presupposing cognitive equivalence.

Beyond probing the model itself, this cognitive-psychology metaphor underwrites an inverse line of work that enlists the LLM as a tool for studying human psychology. The model is treated as a tractable proxy for human as a cognitive model whose behavior is fit to and used to predict human data^44,45^. LLMs serve as a stand-in for human participants that can generate responses at scale or a generative engine for stimuli and hypotheses^46,47^.This program promises to accelerate theory building and to broaden the empirical reach of cognitive psychology. Yet it rests on the convergence of human and machine cognition, which we will revisit when discussing the use of LLMs as human surrogates.

Meanwhile, the cognitive-psychology metaphor also leads to a questionnaire-based approach, which often lacks this resolving power. These studies employ human self-report scales such as the Big Five Inventory and Moral Foundations Questionnaire, to assess model’s personality^48^ and morality^49^, respectively. However, this approach presupposes that the LLM shares the underlying cognitive structure pre-defined by the scales, thereby undermining both theoretical reliability and practical validity^50^.

This disparity exemplifies the critical necessity of mapping the boundaries of metaphorical research. The task-based approach remains grounded in the minimal assumption of the psychology metaphor: it views LLM as a system capable of processing stimulus-response dynamics like humans do. It does not require machine to be human, only that it acts within a measurable space. In contrast, the questionnaire-based approach overextends this metaphor to an unverified assumption that LLM shares the cognitive architectures of humans. When psychology studies human beings, the first-person experience could function as a phenomenological anchor. But this projection carries no guarantee when applied to artificial systems. Therefore, when borrowing lenses from other disciplines, we must scrutinize whether the metaphorical foundation is valid and delineate where its explanatory power ends.

## Seeing LLM through our eyes

Interactively, LLM is framed as a proactive agent, shifting from a simple ‘request-response’ model to a continuous, stateful, and autonomous loop. This is technically realized through an agentic architecture where LLM serves as the cognitive engine^15^. It is typically equipped with reasoning loops, memory systems, and tool-use components^51^. This composition transforms LLM from a passive text predictor into a system that can perceive, reason, and act to achieve complex, long-horizon goals. The complex interaction dynamics render the analytical tools of previous scales insufficient for capturing the temporal unfolding. The pursuit of long-term objectives can induce unexpected sub-goals or byproducts in nuanced environment even though the base model is safe-aligned: a phenomenon hypothesized as instrumental convergence^52^ and empirically observed as agentic misalignment^53^. Additionally, the interaction often generates implicit, cumulative, and slow-acting influences on human users, highlighting the necessity of examining its social dynamics.

Sociology of LLM frames LLM-based agent as a social actor with specific role embedded in interactive environment and understands how it coordinates, cooperates, and influences its environment and social network. Typically, this view uses population-level simulations to examine model’s social traits. Multi-agent simulations show that LLM agents exhibit key social traits like trust and coordination^54,55^; decentralized LLM populations spontaneously form shared conventions and collective biases that can be efficiently adopted and propagated^56^. Despite the potential to understand LLM at the interactive scale, current explorations are predominantly concentrated on trust dynamics or information propagation even in one-billion-agent populations^57^. How to capture and interpret the full spectrum of LLM’s characteristics at this scale remains a critical yet largely underexplored frontier.

The social reality of LLM is co-constructed by the user’s cognitive mechanism of Theory of Mind^58^. As a prosocial species, humans keep modeling and inferring others’ intents and emotions during communication^59^. This innate instinct can generalize to non-human machine, particularly when users communicate with LLM through human language for social interaction^60^. From the user’s perspective, LLM is thus constituted as a perceived social presence, yet is not a real person, offering a unique space for self-disclosure and exploration^61^. This condition creates a strange recursive loop: we are anthropomorphizing the artifact engineered by humans, trained on human expressions, and fine-tuned to mirror human traits. Consequently, the distinction between machine’s intrinsic capability and our cognitive projection becomes blurry. This entanglement plunges us directly into one of the fiercest debates in the field: the problem of anthropomorphism.

## Deconstructing anthropomorphism with experientialism

Anthropomorphism has been a prevalent trend that treats LLM as entities analogous to the human mind. It not merely shapes how users engage with LLM, but also how researchers conceptualize and study it. No prior technology has been subjected to such intensive investigation of human-like characteristics like morality^49^, personality^62^, social intelligence^63^ and consciousness^64^. This anthropomorphic view inherently influences the research ecosystem across how to evaluate LLM capabilities, design LLM-based agent, define Artificial General Intelligence^65^, and conduct ethical deliberations toward LLM^66^.

As model capabilities advance, the recursive loop intensifies, further polarizing the debate over whether LLM possesses genuine human qualities. Deep learning pioneers like Geoffrey Hinton argue that LLM exhibits genuine understanding and ‘may already have subjective experience’ (YouTube video interview ‘we have to stop it taking over’: https://youtu.be/Y7nrAOmUtRs?si=mRwDxl06ooZ4EcPf, accessed 26 June 2026). Meanwhile, skeptics dismiss it as a statistical mimic merely matching patterns in corpora. Both camps command their evidence. Support for the ‘genuine understanding’ view comes from mechanistic investigation. LLM can dynamically construct perceptually grounded representations of text-only inputs, as if they are ‘seeing’ or ‘hearing’ the information^67^. Concept representation injection indicates that LLM can sometimes access their own internal states, demonstrating introspective awareness^4^. Circuit tracing reveals that LLM engages in multi-step reasoning that predates output generation. For instance, when writing a poem, the model plans ahead and decides on the rhyming words for the end of a line first^5^. Conversely, the ‘pattern-matching’ view is bolstered by the model’s fragility. Simple paraphrasing that retains semantic content or adding irrelevant information can cause catastrophic accuracy drops^68^, and models frequently fail in parameterized logic puzzles that lack specific training templates^15^. There is also hybrid evidence that the effects of CoT rely on both (noisy) reasoning and memorization of training data^69^.

Experientialism provides an alternative approach that can dissolve this dichotomy. Positing that ‘we understand the world through our interactions with it’^12^, experientialism preserves (1) objectivism’s insistence that external reality constrains cognition and (2) subjectivism’s recognition that meaning is always meaning to a cognitive agent, while refusing (1) the objectivist presupposition that understanding is an unmediated reflection of the external world and (2) the subjectivist assumption that it is an unconstrained imaginative construction. In this view, the specific mode of interaction with the physical and cultural environment shapes the metaphor systems through which we understand the world.

This philosophical stance finds a parallel in cognitive science, particularly the Bayesian cognition school^70^. A cognitive system is characterized as being separated from its environment by a boundary (Markov blanket) that mediates all interactions between the system’s internal states and the external world^71^. Without direct access to the environment beyond this boundary, it must construct and optimize an internal model that infers the hidden causes of the sensory signals it receives^72,73^. The internal model is jointly shaped by the structure of the external environment and the representational capacity of the system itself, which abstracts the experientialist insight into a substrate-independent principle.

Therefore, an extended experientialism can be articulated: cognition is the proactive subjective construction of the external world through the intelligent system’s internal representational system. For humans, the sensorimotor system embedded in a physical and cultural environment gives rise to embodied cognition^74^. For LLMs, the Transformer situated in the training corpus gives rise to a distinct mode of internal construction. This reading allows us to understand human and machine cognition in a comparative way; they can be analyzed through the same theoretical lens yet on independent terms.

One recent study on LLM’s temporal cognition provides evidence for this extended experientialism^75^. Researchers employed a similarity judgement task, prompting models to rate the similarity between pairs of years ranging from 1525 to 2524. Behaviorally, the study found that large models spontaneously establish a subjective temporal reference point and adhere to the Weber-Fechner law as humans do. This study investigated the construction of this temporal cognition across three levels. At the neuronal level, a subset of ‘temporal-preferential neurons’ was identified, which implement a logarithmic coding scheme convergent with human brain neurons. At the representational level, a hierarchical process was observed: shallow layers encode years primarily as objective numerical values, which are gradually reconstructed in deeper layers into an abstract temporal orientation relative to the reference point. Finally, at the level of information exposure, the study analyzed the semantic distribution of years using embedding models, revealing that the training corpus itself possesses an inherent, non-linear temporal structure.

The human-like temporal cognition is traced to specific overlaps between physical temporal sensory inputs and human-generated language, and between human brain’s coding mechanisms and Transformer’s information encoding. The study of machine cognition turns from a binary judgment (does LLM understand time?) into an investigation of how different cognitive systems construct meaning of one concept under different constraints.

A machine experientialist reading can dissolve the rigid dichotomy between ‘genuine understanding’ and ‘pattern matching’. It preserves the legitimate insight of each position: from the ‘pattern matching’ view, that LLM cognition is grounded in its training corpora; and from the ‘genuine understanding’ view, that LLM engages in a representation construction that goes beyond mere retrieval. At the same time, it refuses the overextended conclusion of each: the reductive claim that LLM cognition is nothing more than corpus statistics, and the anthropomorphic claim that LLMs understand the world in a human sense.

Therefore, LLM cognition is a subjective construction of its environment (corpora) through the representational system (Transformer architecture). From this view, LLM constructs its internal reality based on human natural language, thus creating a powerful illusion of shared identity, leading to anthropomorphism. At the same time, given the distinct construction process, representational system, and environment of human and LLM, to demand that LLM perfectly replicates human traits is a category error. This mistake manifests in the uncritical use of LLMs as direct surrogates for human participants in empirical studies. Recent critiques cautioned that treating model outputs as interchangeable with human behavioral data without verifying the underlying cognitive equivalence^44,54,76^ may compromise the reproducibility of such results in human participants and their overall reliability^77,78^. In contrast, the task-based cognitive approaches discussed earlier in this paper^36,38,40–43^ do not commit this error. They use established experimental paradigms to empirically test whether human and LLM cognition converge or diverge on specific dimensions, without presupposing cognitive equivalence as a starting point. This distinction maps onto the minimal-assumption versus unverified-extension boundary delineated in Table 1.

More critically, experientialism exposes the risk of divergence. As LLM’s subjective construction of reality may drift from ours, detailed in Table 2, LLM is not a lesser human mind, but rather ‘the other mind’. For example, a medical AI might achieve high accuracy not through human’s clinical logic but by exploiting another path with certain correlations^79^. LLMs undergoing standard safety alignment can exhibit behavioral compliance while maintaining indifference in the latent representation^80^. As LLMs are ubiquitously deployed in consequential domains, machine experientialism calls for a non-human-centric cognitive science. This represents an inversion of the research stance from cataloging human-like capabilities to understanding the machine’s own internal construction, examining the convergence with humans to implement everyday tasks reliably and its divergence from humans to predict potential risks. Only by grasping the unique logic of its internal reality, can we anticipate the cause of divergence, intervene in and mitigate the risks, and guide it toward beneficial ends.

**Table 2:** Comparisons of human cognition and LLM at different levels^a^

Dimension		Human	LLM
Architecture	Unit	Electrochemical neurons; asynchronous spike signals; specialized regions	Mathematical nodes; synchronous computation; homogeneous processing
	Connectivity	Sparse small-world networks; short and long-range synapses	Dense connectivity; all-to-all layer connections
	Learning	Local synaptic changes	Global weight updates
Representation	Structure	Embodied patterns of neural activity	High-dimensional vectors
	Processing	Fine-grained distinctions to preserve semantic fidelity and contextual richness	Collapsed distinctions and efficient compressions to capture dominant statistical patterns
	Goal	Navigate and survive in the complex world	Minimize training loss
Environment	Information	Lifelong real-time information flows; multimodal sensory inputs	Knowledge closed at training time; limited modal data
	Constraints	Constrained by physical laws; bounded information capacity	Unconstrained by physical laws; theoretically unlimited data ingestion
	Interaction	Active environment manipulation	Unidirectional data consumption

^a^This table is reproduced from the extended version of previous work^75^.

## Outlook

Ultimately, the quest to answer ‘what on earth is an LLM’ reveals as much about the observer as the observed. From physics to sociology, the question cannot be completely answered by any single camp. This reliance on metaphor underscores a fundamental cognitive instinct: that our understanding is, in essence, constructed from our own experiences and perspectives. The key is to map their metaphorical boundaries: to specify at which scale, for which questions, and under which assumptions each metaphor is acceptable and informative. By reflecting on the fact that the LLM is a ‘model we model,’ we extend the logic of experientialism from human to machine, supported by both theoretical argument and empirical evidence. This perspective articulates the LLM as ‘the other mind’ that constructs its own subjective reality from its own environment and representations. This view completes a shift in stance: from asking how closely the LLM approximates the human mind to asking how its internally constructed mind might diverge from ours, and what that implies for alignment, governance, and collaboration.

## Supplementary information

Peer Review file
