# Supplementary material for: Understanding large language models demands distinguishing human projection from machine cognition
Source: Commun Psychol. 2026 Jul 16;4:108. doi: 10.1038/s44271-026-00508-6 (PMC13376899; doi:10.1038/s44271-026-00508-6)
Supplement: Supplementary file 1 — Peer Review file [file 44271_2026_508_MOESM1_ESM.pdf]

nature portfolio

Peer Review File

Understanding large language models demands distinguishing human projection from machine cognition

Corresponding Author: Professor Yan Teng

This file contains all editorial decision letters in order by version, followed by all author rebuttals in order by version.

Version 0:

Decision Letter:

** Please ensure you delete the link to your author homepage in this e-mail if you wish to forward it to your coauthors **

Dear Professor Teng,

Thank you for your patience during the peer-review process. Your manuscript titled "Understanding large language models demands distinguishing human projection from machine cognition" has now been seen by 2 reviewers, and I include their comments at the end of this message.

The reviewers are in principle enthusiastic about your work. However, they also mention a number of concerns. Many of the concerns they raise are aligned with the editorial assessment of your paper. We are very interested in the possibility of publishing your manuscript in Communications Psychology, but would like to consider a revised manuscript before we make a decision on publication.

For a Perspective to be suitable for publication in Communications Psychology, we expect the piece to build on a fair and up-to-date overview of the relevant literature and to put forward a strong, original proposal against this backdrop. Because the journal serves psychologists and researchers in closely related disciplines, we expect these pieces to be informed by current research in psychology and to clearly convey the proposal's relevance to the field.

We therefore ask you to address the reviewers' concerns by developing the conceptual framework of extended experientialism more clearly. Revisions are further needed to address questions regarding interpretations of LLM vs human minds, and the dichotomy between genuine understanding and pattern matching.

EDITORIAL POLICIES AND FORMATTING

You will find a complete list of formatting requirements following this link: https://www.nature.com/documents/commsj-style-formatting-checklist-review-perspective.pdf
Please use the checklist to prepare your manuscript for resubmission.

* TRANSPARENT PEER REVIEW: Communications Psychology uses a transparent peer review system. This means that we publish the editorial decision letters including Reviewers' comments to the authors and the author rebuttal letters online as a supplementary peer review file. We publish these records for all accepted manuscripts. However, on author request, confidential information and data can be removed from the published reviewer reports and rebuttal letters prior to publication. If your manuscript has been previously reviewed at another journal, those Reviewers' comments would not form part of the published peer review file.

If you have any questions about any of our policies or formatting, please don't hesitate to contact me.

Please use the following link to submit your revised manuscript and a point-by-point response to the referees’ comments (which should be in a separate document to any cover letter):
Link Redacted

** This url links to your confidential home page and associated information about manuscripts you may have submitted or be reviewing for us. If you wish to forward this email to co-authors, please delete the link to your homepage first **

We hope to receive your revised paper within 12 weeks; please let us know if you aren't able to submit it within this time so that we can discuss how best to proceed. If we don't hear from you, and the revision process takes significantly longer, we may close your file.

We understand that due to the current global situation, the time required for revision may be longer than usual. We would appreciate it if you could keep us informed about an estimated timescale for resubmission, to facilitate our planning. Of course, if you are unable to estimate, we are happy to accommodate necessary extensions nevertheless.

Please do not hesitate to contact me if you have any questions or would like to discuss these revisions further. We look forward to seeing the revised manuscript and thank you for the opportunity to review your work.

Best regards,

Troby Lui, PhD
Associate Editor
Communications Psychology

REVIEWERS' EXPERTISE:

Reviewer #1: AI, philosophy of science
Reviewer #2: AI, cognitive science

REVIEWERS' COMMENTS:

Reviewer #1 (Remarks to the Author):

The paper is admirable for its thoughtful discussion of the many conceptual and scientific challenges facing understanding large language models (LLMs). The authors propose "extended experientialism" as a better way to think about LLMs, suggesting that "the model constructs a unique internal reality from its own architecture and environment." The core idea is that the metaphors we use to describe LLMs are often partial (corresponding to a small cross section of LLM), and we need to avoid anthropomorphism, or thinking of LLM as a human mind.

There is much value in the conceptual framework in this paper. I want to raise a few questions and concerns that I think the authors should be able to address, with some revisions and expansions.

First, what is this core position called "extended experientialism"? The authors suggest it should be a middle path between objectivism and subjectivism, and it corresponds to the idea that meaning, truth, and rationality emerge from the interaction between our embodied selves and the world we live. But on the face of it, such a position is compatible with objectivism, and also with subjectivism. The discussions later on in the paper did not spell out how this "emergence" through interaction differs from standard positions about objectivism or subjectivism. The paper needs to be clearer on what this position amounts to exactly.

Second, the difficulties the paper raises regarding various metaphors for interpreting LLMs also arises for interpreting human beings. We have neuroscience techniques, physics tools, and biology concepts. All of them are scientific disciplines studying human beings and human cognition. Are they also partial? If so, are such scientific disciplines merely doing metaphorical work on trying to understand humans (but failing)? The worry here is that the authors' argument may prove too much. We are applying familiar techniques from various disciplines and transferring them from studying humans to studying LLMs. Yes, LLMs are different from humans. But at the same time, isn't there something in common between LLMs and humans, such that it warrants such a transfer of tools and concepts? The authors should try to address this concern about the validity of their general argument in this paper.

Third, how does the experientialist reading dissolve the dichotomy between genuine understanding and pattern matching? Saying that it is the "subjective construction of its environment through the representational system" does not really get to this question. It describes what LLM does. What is the experientialist reading adding to dissolving the dichotomy? The paragraph around lines 253 to 270 seem to confirm that LLMs do have abstraction and genuine understanding through their representational structure.

The later paragraphs seem to emphasize that LLMs are unique or different from human minds, so we should be cautious about carrying metaphors from humans to LLMs. Yes, that is helpful, but how is this a prediction from the extended experientialism position? One can say the same thing even granting LLMs have genuine understanding, or insisting they are stochastic parrots. The worry here is that it is not clear there is sufficient advantage adopting the "extended experientialism" framework.

I want to emphasize there is much to like in this paper, and it can make a valuable contribution to this literature. It is also admirably interdisciplinary and creative. But the paper, as it is written, can benefit from more conceptual clarity about what is

being proposed, and how it leads to a better understanding of LLMs.

Reviewer #2 (Remarks to the Author):

Summary

The manuscript "Understanding large language models demands distinguishing human projection from machine cognition" presents a conceptual perspective on how researchers interpret the behavior of large language models (LLMs). Drawing on Lakoff and Johnson's theory of conceptual metaphors, the author(s) argue that many current approaches to understanding LLMs rely on metaphorical projections from established disciplines. They identify four prominent metaphorical lenses: physics, neuroscience, psychology, and sociology, and organize them across three levels of analysis: mechanistic, behavioral, and interactive. The manuscript argues that these metaphors illuminate certain aspects of LLM behavior while simultaneously obscuring others, leading to fragmented and sometimes misleading interpretations. To move beyond the debate over whether LLMs demonstrate "genuine understanding" or merely perform "pattern matching", the authors introduce the notion of "machine experientialism". In this framework, "LLM cognition" is conceptualized as the system's internal construction of reality based on its architecture and training data. The authors conclude that researchers should shift away from evaluating LLMs in terms of their similarity to human cognition and instead focus on understanding how these systems construct their own internal representations and behavioral regularities.

Major points

The manuscript raises an interesting (and potentially timely) conceptual question about how researchers theorize about LLM cognition. The attempt to synthesize perspectives from different disciplines and to reflect on the role of metaphor in scientific reasoning is potentially valuable. However, several aspects of the argument require clarification or stronger engagement with the existing literature.

First, the central claim that interdisciplinary approaches to LLMs are primarily metaphorical is presented in a way that risks oversimplifying the methodological status of several research programs. Approaches described as physics, neuroscience, or psychology metaphors often involve formal models, empirical analyses, or experimentally grounded paradigms rather than merely analogical reasoning. For example, mechanistic interpretability research attempts to identify computational structures within models, and statistical physics approaches often derive mathematically grounded descriptions of training dynamics. Treating these approaches mainly as metaphorical projections may therefore mischaracterize their epistemic role. A clearer distinction between metaphor as a conceptual heuristic and formal explanatory modeling would strengthen the manuscript.

Second, the proposed concept of "machine experientialism" is presented as the main theoretical contribution, yet it remains somewhat underdeveloped. Although the author(s) frame it as a way to move beyond the dichotomy between “genuine understanding” and “pattern matching,” the framework is primarily articulated at a philosophical level. It remains unclear how adopting this perspective would concretely guide empirical research or generate new testable predictions about LLM behavior. The brief connection to Bayesian cognition and active inference is intriguing but not developed sufficiently to anchor the proposal within established cognitive science theory.

Third, the manuscript’s treatment of psychological research on LLMs appears somewhat narrow (and potentially outdated). The authors suggest that the psychology metaphor often leads to questionnaire-based approaches that attribute human-like traits such as personality or morality to LLMs. While such studies exist and may indeed raise concerns about anthropomorphic interpretation, this characterization overlooks a substantial and rapidly growing body of experimental work that applies established cognitive paradigms to LLMs. For example, recent research has used metamemory paradigms such as judgments of learning to compare human and LLM cognition and has demonstrated systematic differences in metacognitive monitoring between the two (Huff & Ulakci, 2025). Similarly, other studies use reasoning tasks, perception-like judgments, or decision paradigms drawn from cognitive psychology (Uhler et al., 2026). These approaches do not rely on questionnaire-style anthropomorphism but instead employ experimentally grounded behavioral methods. As a result, the manuscript may partially reflect an earlier stage of the literature and would benefit from acknowledging the broader experimental tradition emerging in "machine psychology".

Finally, the manuscript positions itself as a synthesis across disciplines, yet the literature coverage is somewhat imbalanced. Several references come from preprints or informal sources, while some relevant peer-reviewed work in cognitive science and psychology is not discussed. A more systematic engagement with this literature would strengthen the conceptual argument and situate the proposed framework within ongoing debates about LLM cognition.

Minor points

The balance between sections could be improved. The mechanistic perspectives receive relatively detailed discussion, whereas the behavioral and interactive perspectives are treated more briefly. Given that the central argument concerns the comparison of disciplinary lenses, a more balanced approach would strengthen the paper's structure.

Regarding open science practices, the manuscript does not address transparency, reproducibility, or accessibility in LLM research. Given the increasing reliance on proprietary models (e.g., OpenAI's ChatGPT) and opaque training pipelines, these issues are highly relevant to interpreting and evaluating claims about model cognition. A brief discussion of these challenges would strengthen the broader methodological perspective.

References mentioned in the review

Huff, M., & Ulakci, E. (2025). Judgments of learning distinguish humans from large language models in predicting memory. Scientific Reports, 15(1), 35030. https://doi.org/10.1038/s41598-025-22290-x

Uhler, L., Jordan, V., Buder, J., Huff, M., & Papenmeier, F. (2026). Influence of solution efficiency and valence of instruction on additive and subtractive solution strategies in humans, GPT-4, and GPT-4o. Communications Psychology, 4(1), 41. https://doi.org/10.1038/s44271-026-00403-0

** Visit Nature Research's author and referees' website at www.nature.com/authors for information about policies, services and author benefits**

Communications Psychology is committed to improving transparency in authorship. As part of our efforts in this direction, we are now requesting that all authors identified as ‘corresponding author’ create and link their Open Researcher and Contributor Identifier (ORCID) with their account on the Manuscript Tracking System prior to acceptance. ORCID helps the scientific community achieve unambiguous attribution of all scholarly contributions. You can create and link your ORCID from the home page of the Manuscript Tracking System by clicking on ‘Modify my Springer Nature account’ and following the instructions in the link below. Please also inform all co-authors that they can add their ORCIDs to their accounts and that they must do so prior to acceptance.
https://www.springernature.com/gp/researchers/orcid/orcid-for-nature-research

For more information please visit http://www.springernature.com/orcid

If you experience problems in linking your ORCID, please contact the Platform Support Helpdesk.

Version 1:

Decision Letter:

** Please ensure you delete the link to your author homepage in this e-mail if you wish to forward it to your coauthors **

Dear Professor Teng,

Thank you for your patience during the peer-review process. Your manuscript titled "Understanding large language models demands distinguishing human projection from machine cognition" has now been seen by 2 reviewers, and I include their comments at the end of this message.

The reviewers are in principle enthusiastic about your work. However, they also mention a number of concerns.We are very interested in the possibility of publishing your manuscript in Communications Psychology, but would like to consider your response to these concerns in the form of a revised manuscript before we make a decision on publication.

To aid you with that task, I have included a marked-up version of your manuscript. Editorially, we consider it crucial that the reviewers remaining concerns regarding the potential self-reference problem, clarification on the claim regarding machine experientialism, as well as the discussion on the use of LLM in psychological research are carefully addressed in the revised manuscript.

In sum, we invite you to revise your manuscript taking into account all reviewer comments.

EDITORIAL POLICIES AND FORMATTING

You will find a complete list of formatting requirements following this link: https://www.nature.com/documents/commsj-style-formatting-checklist-review-perspective.pdf
Please use the checklist to prepare your manuscript for resubmission.

* TRANSPARENT PEER REVIEW: Communications Psychology uses a transparent peer review system. This means that we publish the editorial decision letters including Reviewers' comments to the authors and the author rebuttal letters online as a supplementary peer review file. We publish these records for all accepted manuscripts. However, on author request, confidential information and data can be removed from the published reviewer reports and rebuttal letters prior to publication.

If your manuscript has been previously reviewed at another journal, those Reviewers' comments would not form part of the published peer review file.

If you have any questions about any of our policies or formatting, please don't hesitate to contact me.

Please use the following link to submit your revised manuscript and a point-by-point response to the referees' comments (which should be in a separate document to any cover letter):
Link Redacted

** This url links to your confidential home page and associated information about manuscripts you may have submitted or be reviewing for us. If you wish to forward this email to co-authors, please delete the link to your homepage first **

We hope to receive your revised paper within 12 weeks; please let us know if you aren't able to submit it within this time so that we can discuss how best to proceed. If we don't hear from you, and the revision process takes significantly longer, we may close your file.

We understand that due to the current global situation, the time required for revision may be longer than usual. We would appreciate it if you could keep us informed about an estimated timescale for resubmission, to facilitate our planning. Of course, if you are unable to estimate, we are happy to accommodate necessary extensions nevertheless.

Please do not hesitate to contact me if you have any questions or would like to discuss these revisions further. We look forward to seeing the revised manuscript and thank you for the opportunity to review your work.

Best regards,

Troby Lui, PhD
Associate Editor
Communications Psychology

REVIEWERS' COMMENTS:

Reviewer #1 (Remarks to the Author):

I thank the authors for revising the manuscript and responding to my comments and suggestions. The arguments and ideas are clearer now.

One small issue I still have trouble understanding concerns the claim that "machine experierentialism" commits one to "refusing the objectivist presupposition of mind-independent truth and the subjectivist assumption of unconstrained imaginative construction." The authors write at line 85, "This cognitive process advances the philosophy of experientialism, a middle path between objectivism and subjectivism, claiming that meaning, truth, and rationality are neither properties of the world nor arbitrary creations of our minds. Instead, they emerge from the interaction between our embodied selves and the world we live." This strikes me as a very bold claim, especially if it is meant to reject the existence of mind-independent truths or properties of the world. I wonder whether the authors really intend to endorse such a strong position. One can hold that machines interact with the world differently from the way humans do while still accepting that there are mind-independent facts about the world. My worry is that denying mind-independent facts may unnecessarily weaken the authors' argument.

There is also a familiar self-reference problem here. Is the authors' claim about machine experientialism itself meant to be a true claim about the actual world, perhaps corresponding to a scientific fact that holds independently of our perspectives? If so, then it seems to presuppose at least some mind-independent facts (e.g. the authors' claim). I therefore encourage the authors to clarify whether they mean to reject mind-independent truth altogether, or only to reject a particular objectivist conception of meaning.

Reviewer #2 (Remarks to the Author):

The authors have made a constructive effort to address the previous comments, and the manuscript has improved in clarity, particularly regarding the conceptual positioning of experientialism. However, a central concern remains unresolved: the characterization of psychological approaches to LLMs.

Although the manuscript briefly acknowledges task-based approaches, their role is underemphasized relative to questionnaire-based studies. This creates an imbalanced picture of the field and risks giving readers the impression that psychological research on LLMs is primarily anthropomorphic in nature. Given that the manuscript positions itself as a cross-disciplinary synthesis, a more balanced treatment of experimental cognitive approaches is necessary.

This imbalance is especially evident in the paragraph discussing the use of LLMs as surrogates for human participants. The

manuscript states that this "mistake is already spreading to psychology, sociology, and economics," raising concerns about reproducibility and reliability. While these concerns are important and well-motivated, the current formulation risks overstating the extent and nature of the problem. In particular, it does not sufficiently distinguish between uncritical uses of LLMs as human substitutes and more recent, theoretically grounded approaches that explicitly investigate where LLM behavior converges with or diverges from human cognition (e.g., Huff & Ulakci, 2025; https://www.nature.com/articles/s41598-025-22290-x). In cognitive psychology, LLMs are increasingly used not as surrogates in a strong sense, but as tools for probing cognitive processes under controlled conditions. These approaches directly address the concerns raised by the authors, rather than exemplifying the "mistake" described. Clarifying this distinction would make the argument more precise and avoid giving the impression that current research practices are overly generalized.

A substantial (and growing) body of work applies established experimental paradigms from cognitive psychology to LLMs, including similarity judgments, decision-making tasks, and metacognitive paradigms. These approaches do not rely on anthropomorphic assumptions but instead use behavioral methods to generate falsifiable comparisons between human and model cognition. For example, recent work using metamemory paradigms demonstrates systematic differences in metacognitive monitoring between humans and LLMs.

Addressing this point would substantially strengthen the manuscript's central argument by ensuring that the critique of "metaphor" is directed at specific methodological assumptions rather than at psychological approaches more broadly.

** Visit Nature Research's author and referees' website at www.nature.com/authors for information about policies, services and author benefits**

Communications Psychology is committed to improving transparency in authorship. As part of our efforts in this direction, we are now requesting that all authors identified as ‘corresponding author’ create and link their Open Researcher and Contributor Identifier (ORCID) with their account on the Manuscript Tracking System prior to acceptance. ORCID helps the scientific community achieve unambiguous attribution of all scholarly contributions. You can create and link your ORCID from the home page of the Manuscript Tracking System by clicking on ‘Modify my Springer Nature account’ and following the instructions in the link below. Please also inform all co-authors that they can add their ORCIDs to their accounts and that they must do so prior to acceptance.
https://www.springernature.com/gp/researchers/orcid/orcid-for-nature-research

For more information please visit http://www.springernature.com/orcid

If you experience problems in linking your ORCID, please contact the Platform Support Helpdesk.

Version 2:

Decision Letter:

** Please ensure you delete the link to your author homepage in this e-mail if you wish to forward it to your coauthors **

Dear Professor Teng,

Thank you for submitting your manuscript titled "Understanding large language models demands distinguishing human projection from machine cognition" to Communications Psychology. We have given the paper our careful consideration and appreciate that you provided a rebuttal for the referees' comments. However, the editorial comments provided on the previous version of the manuscript remain unaddressed in the revised version.

We cannot take the revision forward until edits to address the editorial concerns have been fully incorporated. To help you with that task, I have attached the copy of the manuscript including editors' comments.

We would like to invite you to revise your manuscript to address these concerns before we make a final determination on whether to send your manuscript for external review.

We shall hope to receive your revised version as soon as you are able to complete the suggested revisions. If something similar is published in the interim we will have to consider the impact it has on the novelty of a revised manuscript.

If you anticipate a delay of more than four weeks, please let us know. Should your manuscript be substantially delayed without notifying us in advance and your article is eventually published, the received date may be that of the revised, not the original, version.

If you are not interested in submitting a suitably revised manuscript in the future please let me know immediately so we can close your file. If you have any questions, please contact me.

Please use the link below when you are prepared to resubmit.
Link Redacted

Thank you for your interest in Communications Psychology.

Best regards,
Troby Lui

Troby Lui, PhD
Associate Editor
Communications Psychology

Version 3:

Decision Letter:

** Please ensure you delete the link to your author homepage in this e-mail if you wish to forward it to your coauthors **

Dear Professor Teng,

Thank you for your patience while we evaluated your revisions. Your manuscript titled "Understanding large language models demands distinguishing human projection from machine cognition" has now been assessed editorially. We remain interested in the possibility of publishing your manuscript in Communications Psychology; however, critical issues from the previous round need to be addressed for us to make a final decision on publication.

In particular, the manuscript is still lacking in its discussion of LLMs as a tool in psychological research; editorially, we consider this consider a necessary element of the paper, and a narrowing of its scope is not desirable. To aid you in addressing this request, I have included a marked-up version of your manuscript.

* TRANSPARENT PEER REVIEW: Communications Psychology uses a transparent peer review system. This means that we publish the editorial decision letters including Reviewers' comments to the authors and the author rebuttal letters online as a supplementary peer review file. We publish these records for all accepted manuscripts. However, on author request, confidential information and data can be removed from the published reviewer reports and rebuttal letters prior to publication. If your manuscript has been previously reviewed at another journal, those Reviewers' comments would not form part of the published peer review file.

If you have any questions about any of our policies or formatting, please don't hesitate to contact me.

Please use the following link to submit your revised manuscript:
Link Redacted

** This url links to your confidential home page and associated information about manuscripts you may have submitted or be reviewing for us. If you wish to forward this email to co-authors, please delete the link to your homepage first **

We hope to receive your revised paper within 4 weeks; please let us know if you aren’t able to submit it within this time so that we can discuss how best to proceed. If we don’t hear from you, and the revision process takes significantly longer, we may close your file.

Please do not hesitate to contact me if you have any questions or would like to discuss these revisions further. We look forward to seeing the revised manuscript and thank you for the opportunity to review your work.

Best regards,

Troby Lui, PhD
Associate Editor
Communications Psychology

** Visit Nature Research's author and referees' website at www.nature.com/authors for information about policies, services and author benefits**

Communications Psychology is committed to improving transparency in authorship. As part of our efforts in this direction, we are now requesting that all authors identified as ‘corresponding author’ create and link their Open Researcher and Contributor Identifier (ORCID) with their account on the Manuscript Tracking System prior to acceptance. ORCID helps the scientific community achieve unambiguous attribution of all scholarly contributions. You can create and link your ORCID from the home page of the Manuscript Tracking System by clicking on ‘Modify my Springer Nature account’ and following the instructions in the link below. Please also inform all co-authors that they can add their ORCIDs to their accounts and that they must do so prior to acceptance.
https://www.springernature.com/gp/researchers/orcid/orcid-for-nature-research

For more information please visit http://www.springernature.com/orcid

If you experience problems in linking your ORCID, please contact the Platform Support Helpdesk.

Open Access This Peer Review File is licensed under a Creative Commons Attribution 4.0 International License, which permits use, sharing, adaptation, distribution and reproduction in any medium or format, as long as you give appropriate credit to the original author(s) and the source, provide a link to the Creative Commons license, and indicate if changes were made.
In cases where reviewers are anonymous, credit should be given to 'Anonymous Referee' and the source.
The images or other third party material in this Peer Review File are included in the article’s Creative Commons license, unless indicated otherwise in a credit line to the material. If material is not included in the article’s Creative Commons license and your intended use is not permitted by statutory regulation or exceeds the permitted use, you will need to obtain permission directly from the copyright holder.
To view a copy of this license, visit https://creativecommons.org/licenses/by/4.0/

Dear Reviewer 1,

We sincerely thank you for your careful reading and thoughtful comments. Your questions have pushed us to sharpen the theoretical foundations of our argument considerably. We have revised our manuscript in response to your four comments. We detail our point-by-point responses below. All changes are tracked using change mode in the revised manuscript.

Reviewer's Comment #1

First, what is this core position called "extended experientialism"? The authors suggest it should be a middle path between objectivism and subjectivism, and it corresponds to the idea that meaning, truth, and rationality emerge from the interaction between our embodied selves and the world we live. But on the face of it, such a position is compatible with objectivism, and also with subjectivism. The discussions later on in the paper did not spell out how this "emergence" through interaction differs from standard positions about objectivism or subjectivism. The paper needs to be clearer on what this position amounts to exactly.

Response #1

We thank the reviewer for this incisive observation. We agree that our original framing of experientialism as a “third path” was imprecise and potentially misleading, as it suggests a position that stands apart from objectivism and subjectivism. We have revised the relevant section to clarify the following two points.

1. **Exprientialism itself is a powerful alternative to objectivism vs. subjectivism**

   The reviewer is correct that the extended experientialism feels compatible with aspects of both positions; indeed, this is precisely the theoretical strength of experientialism itself. As Lakoff and Johnson articulate in Chapter 29 of *Metaphors We Live By*, experientialism does not reject objectivism and subjectivism wholesale. Rather, it (1) preserves the legitimate concerns motivating each, including objectivism's emphasis on external constraints and subjectivism's emphasis on individual meaning construction; and (2) refuses the over-extended commitment of each, including the objectivist presupposition of absolute, mind-independent truth, and the subjectivist insistence that imaginative understanding is entirely unconstrained.

   In this sense, it *dissolves* the ‘stochastic parrot’ vs. ‘genuine understanding’dichotomy in the same manner. It (1) preserves that, from the “stochastic parrot” position, LLM cognition is grounded in its training corpora; and, from the “genuine understanding” position, LLMs are engaged in a form of subjective construction; (2) refuses both the reductive claim that LLM cognition is nothing more than corpus retrieval, and the anthropomorphic claim that LLMs understand the world in a genuinely human sense. The conclusion is that LLMs leverages their specific representational architecture

(Transformer) to construct their own systematic interpretation of the training corpora (We elaborate on this point further in our response to Comment #3.)

Revision 1: we replaced the original sentence "Experientialism provides a third path" to avoid implying that experientialism is a position parallel to objectivism and objectivism:

"Experientialism provides an alternative approach that can dissolve this dichotomy. Lakoff and Johnson …"

Revision 2: we added the characterization of how experientialism serves as a powerful alternative between obejctivism and subjectivism:

"(Experientialism) dissolves the long-standing debate between objectivism and subjectivism by (1) preserving the objectivism's insistence that external reality constrains cognition and subjectivism's recognition that meaning is always meaning to a cognitive agent, while (2) refusing the objectivist presupposition of mind-independent truth and the subjectivist assumption of unconstrained imaginative construction. In this view, the specific mode of interaction with the physical and cultural environment shapes the metaphor systems through which we make sense of reality."

2. What does "Extended" Experientialism extend?

In Lakoff and Johnson's original formulation, human cognition emerges from the interaction with physical world through body. Based on subsequent developments in cognitive science, our extension generalizes this concept of embodiment in human to a broader notion of representational architecture that applies to both human and intelligent machines. In particular, the Bayesian cognition theory abstracts the experientialist insight into a substrate-independent principle. It reconceptualize cogniton as the process by which an agent builds and refines generative models of its environment to minimize prediction error. This generalization provides a useful framework for understanding machine cognition and distinguishing human projection.

Revision 3: although our original manuscript briefly noted the connection to Bayesian cognition, we acknowledge that this connection was not elaborated sufficiently. We added the following elaboration to anchor our "extended" experientialism:

"This philosophical stance finds a parallel in cognitive science, particularly the Bayesian cognition school [63]. A cognitive system is characterized as being separated from its environment by a boundary (Markov blanket) that mediates all interactions between the system's internal states and the external world [64]. Without direct access to the environment beyond this boundary, it must construct and optimize an internal model that infers the hidden causes of the sensory signals it receives [65,66]. The internal model is jointly shaped by the structure of the external environment and the representational capacity of the system itself, which abstracts the experientialist insight into a substrate-independent principle.

Therefore, an extended experientialism can be articulated: cognition is the proactive subjective construction of the external world through the intelligent system's internal representational system. For humans, the sensorimotor system embedded in a physical and cultural environment gives rise to the embodied cognition that Lakoff and Johnson describe. For LLMs, the Transformer situated in the training corpus gives rise to a distinct mode of internal construction. This reading allows us to understand human and machine cognition in a comparative way; they can be analyzed through the same theoretical lens yet on independent terms."

Reviewer's Comment #2

Second, the difficulties the paper raises regarding various metaphors for interpreting LLMs also arises for interpreting human beings. We have neuroscience techniques, physics tools, and biology concepts. All of them are scientific disciplines studying human beings and human cognition. Are they also partial? If so, are such scientific disciplines merely doing metaphorical work on trying to understand humans (but failing)? The worry here is that the authors' argument may prove too much. We are applying familiar techniques from various disciplines and transferring them from studying humans to studying LLMs. Yes, LLMs are different from humans. But at the same time, isn't there something in common between LLMs and humans, such that it warrants such a transfer of tools and concepts? The authors should try to address this concern about the validity of their general argument in this paper.

Response #2

We thank the reviewer for this insightful comment. We addressed the risk of proving too much and the legitimacy of transfer of tools and concepts in turn.

1. **Complexity presents an epistemological challenge in science**

   The reviewer is correct that the partiality of any single discipline is shared by interpreting human beings. In fact, this goes to the heart of Complexity Science. Anderson's famous work *More is Different* (1972) demonstrated that each level of complex systems (e.g., market , human mind, or LLMs) exhibits emergent properties irreducible to the laws governing lower levels. The rise of Complexity Science as a field suggests that understanding complex systems demands integration of perspective across scales from different disciplines.

   Our work does not challenge the validity of scientific tools. By decomposing the study of LLMs across three scales (mechanistic, behavioral, and interactive) and examining four interpretive paradigms that operate across them, our paper aims to extract the genuine explanatory contributions of each while identifying where their assumptions overreach. The multi-scale architecture of our analysis is a methodological response to the complexity problem.

What makes interpreting LLMs different from interpreting humans is the applicability of "human projection". When studying human, the first-person experience "human projection" could serve as an implicit anchor, which is a good epistemic resource. When the same concepts are transferred to LLMs, we have no phenomenological access to what it is like to be a Transformer. The very projection that aids human self-understanding may yield unverified, over-extended conclusions. Therefore, we undertake the boundary-drawing presented in Table 1 to identify what is empirically grounded and what remains an anthropomorphic assumption carried over by the metaphor.

Revision 4: to make our work more constructive rather than deconstructive, we revised the relevant section to reframe our critical analysis of existing studies. For example, we remove the expressions such as "failing" with more neutral words. We also added explicit claim:

"… physics, neuroscience, psychology, and sociology, organizing them across mechanistic, behavioral, and interactive scales. We aim to identify the explanatory contribution and theoretical boundary of each, thereby provide an integrated understanding of the LLMs."

Revision 5: we elaborated the difference of understanding LLMs and humans:

"... When psychology studies human beings, the first-human experience could function as a phenomenological anchor. But this projection carries no guarantee when applied to artificial systems. Therefore, when borrowing lenses from other disciplines…**"**

2. **If LLMs are different from humans, what warrants applying human-derived concepts to them?**

As described our Response #1, the extended experientialism posits that two systems share a common cognitive principle: a subjective construction of external environment through representational architecure. It provides a principled basis for cross-system comparison: we can analyze both human and machine cognition through the same theoretical lens while respecting the fact that the specific answers differ fundamentally between the two.

Our case study in the manuscript on temporal cognition instantiates this comparative methodology. This study examined human and LLM cognition along the three dimensions that the extended experientialist framework foregrounds: architecture, environment, and representation. For architecutre, it compared the neural encoding mechanisms in human cortical neurons with those in Transformer attention layers. For environment, it contrasted humans' grounding in physical temporal perception with LLMs' exposure to human-generated textual data. For representation, it utilized the same similarity judgment task to both systems, providing a common behavioral measure that allows direct comparison without presupposing equivalence.

In more capable models, the representational structure of temporal concepts converges toward human-like patterns. This similarity emerges because their training corpora, though different in origin from human perceptual experience, preserve much of the statistical structure that the physical

world imprints on language. In experientialist terms, two distinct architectures operating on two distinct but statistically overlapping environments give rise to representational structures that (partially) converge.

Revision 6: we added the implication of the case study on the cross-system comparison under extended experientialism:

"… This study instantiates the comparative methodology under the machine experientialism. The human-like temporal cognition is traced to specific overlaps between physical temporal sensory and human generated language, and between human brain's coding mechanisms and Transformer's information encoding. The study of machine cognition turns from a binary judgment (does LLM understand time?) into investigation of how different cognitive systems construct meaning of one concept under different constraints."

Reviewer's Comment #3

Third, how does the experientialist reading dissolve the dichotomy between genuine understanding and pattern matching? Saying that it is the "subjective construction of its environment through the representational system" does not really get to this question. It describes what LLM does. What is the experentialist reading adding to dissolving the dichotomy? The paragraph around lines 253 to 270 seem to confirm that LLMs do have abstraction and genuine understanding through their representational structure.

Response #3

We thank the reviewer for this comment. Based on our Response #1 and #2, we now elaborate further on how the extended experientialism dissolve the dichotomy as follows.

As clarified in Response #1, the extended experientialism dissolves the 'stochastic parrot' vs. 'genuine understanding'dichotomy in the same manner through which the experientialism dissolves the objectivism vs. subjectivism dichotomy. They both preserve the legitimate insights while refusing the over-extensions of each side.

And as the comparative methodology proposed by extended experientialism in Response #2, the question "does LLM genuinely understand, or merely match patterns?" is not answered yes or no. LLM cognition is grounded in its training corpus (the stochastic parrot position), and LLMs do engage in a form of systematic, constructive interpretation (the genuine understanding position). What it refuses is the overextended conclusion of each: that LLM cognition is nothing more than retrieval (the stochastic parrot position), or that it constitutes understanding in a genuinely human sense (the genuine understanding position).

The positive yield a shift of research stance. It directs inquiry from “how closely the LLM approximates the human mind” toward “what representational structure does the Transformer construct from its textual environment”. Only by understanding LLMs on their own, we could predict their risks and collaborate with them more efficiently.

Revision 7: the reviewer rightly notes that our original manuscript appears to confirm that LLMs possess both pattern-matching and genuine understanding. We have revised the related content to our position clearer:

“A machine experientialist reading can dissolve the rigid dichotomy between ‘genuine understanding’ and ‘pattern matching’. It preserves the legitimate insight of each position: from the ‘pattern matching’ view, that LLM cognition is grounded in its training corpora; and from the ‘genuine understanding’ view, that LLM engages in a representation construction that goes beyond mere retrieval. At the same time, it refuses the overextended conclusion of each: the reductive claim that LLM cognition is nothing more than corpus statistics, and the anthropomorphic claim that LLMs understand the world in a human sense.

Therefore, LLM cognition is a subjective construction of its environment (corpora) through the representational system (Transformer architecture). From this view, LLM constructs its internal reality based on human natural language, thus creating …”

Reviewer's Comment #4
The later paragraphs seem to emphasize that LLMs are unique or different from human minds, so we should be cautious about carrying metaphors from humans to LLMs. Yes, that is helpful, but how is this a prediction from the extended experientialism position? One can say the same thing even granting LLMs have genuine understanding, or insisting they are stochastic parrots. The worry here is that it is not clear there is sufficient advantage adopting the "extended experientialism" framework.

Response #4

We thank the reviewer for this comment on the practical value of extended experientialism that “stochastic parrots” or “genuine understanding” cannot produce.

The reviewer is correct that cautioning against over-extending human metaphors can be stated from either pole of the dichotomy. We think that extended experientialism provides a nuanced prediction. Because LLM cognition is shaped by the interaction between its specific architecture (Transformer) and its specific environment (training corpora), its representational fidelity to human cognition will vary systematically across domains. It directs researchers to ask: for a given cognitive domain, how much of the relevant structure is preserved in the textual environment, and how does the Transformer's architecture reconstruct it?

As LLMs are ubiquitously deployed in various scenarios, carefully examining their convergence (for implementing everyday tasks reliably) and divergence (for predicting potential risks) with humans becomes more and more important. For example, human morality emerges through embodied social interaction, much of which is partially encoded in language. Recent work found that LLMs, though behaviorally compliant, exhibit an inherent state of moral indifference, thereby vulnerable to long-tail risk such as jailbreaks. Further targeted representational alignment improves model's moral reasoning.

The "genuine understanding" view would predict consistent behavioral and representational alignment and cannot explain the systematic fragility. The "stochasitic parrot" view would predict uniform failure and cannot explain the effects of representational alignment. By our extended experientialism, we can identify how LLMs and humans understand "morality" respectively, thereby leveraging the convergence for productive collaboration and intervening the divergence for targeted alignment.

Revision 8: We have revised the corresponding paragraph to articulate the predictive and practical implications of extended experientialism:

"…a medical AI might achieve high accuracy not through human's clinical logic but by exploiting another path with certain correlations [72]. LLMs undergoing standard safety alignment can exhibit behavioral compliance while maintain indifference in the latent representation [73]. As LLMs are ubiquitously deployed in consequential domains, the machine experientialism calls for a non-human centric cognitive science. This represents an inversion of research stance from cataloging human-like capabilities to understanding the machine's own internal construction, examining the convergence with humans for implementing everyday tasks reliably and divergence from humans for predicting potential risks. Only by grasping the unique logic of its internal reality, can we anticipate the cause of divergence, intervene and mitigate the risks, and guide it toward the beneficial ends."

We hope that these revisions adequately address your concerns. Your comments have been invaluable in helping us refine the conceptual clarity and argumentative rigor of this work. We remain open to any further suggestions you may have.

Dear Reviewer 2,

We sincerely thank the reviewer for the careful reading and constructive feedback. The comments have prompted us to sharpen the theoretical articulation, broaden the literature engagement, and improve the structural clarity of the manuscript. All changes are tracked by change mode in the revised manuscript.

Reviewer's Comment #1

First, the central claim that interdisciplinary approaches to LLMs are primarily metaphorical is presented in a way that risks oversimplifying the methodological status of several research programs. Approaches described as physics, neuroscience, or psychology metaphors often involve formal models, empirical analyses, or experimentally grounded paradigms rather than merely analogical reasoning. For example, mechanistic interpretability research attempts to identify computational structures within models, and statistical physics approaches often derive mathematically grounded descriptions of training dynamics. Treating these approaches mainly as metaphorical projections may therefore mischaracterize their epistemic role. A clearer distinction between metaphor as a conceptual heuristic and formal explanatory modeling would strengthen the manuscript.

Response #1

We thank the reviewer for identifying this ambiguity in our presentation. The reviewer is right that the current manuscript may risk conflating the epistemological status of formal explanatory modeling with that of loose analogical reasoning, which is not our intention. We have revised the relevant sections to clarify the following point.

1. **Metaphor as a basic cognitive mechanism, not mere linguistic analogy**

    As we stated in the original manuscript:

    "Articulated by George Lakoff and Mark Johnson in *Metaphors We Live By* [12], metaphor is not merely figures of speech but also a cognitive tool governing how we think and act."

    Our use of "metaphor" throughout the paper is anchored in Lakoff and Johnson's sense. Metaphor is a basic mechanism of human cognition through which one domain of experience is systematically understood in terms of another. Calling an approach "metaphorical" is therefore a meta-scientific observation about the conceptual scaffolding that organizes a field of inquiry, including its prior commitments, explanatory standards, space of admissible questions, and theoretical boundaries.

    This characterization does not diminish the intellectual rigor or empirical validity of the work conducted within a given framework. For example, mechanistic interpretability research, by treating the LLM as an information-processing network whose internal components can be mapped to functional roles, has identified genuine computational structures such as neurons, features, and

circuits. Our contribution is to analyze the scaffoldings that make such discoveries possible and legible, while also identifying where each framework's explanatory reach has its limits.

We realize that this technical usage was not made sufficiently explicit in the original manuscript. Readers unfamiliar with Lakoff and Johnson's theory may interpret “metaphorical” as synonymous with merely analogical or informal. We have made two revisions to prevent this misreading.

Revision 1: we have retitled the Section 2 from “Why do interdisciplinary metaphors work?” to:

> “Interdisciplinary metaphors as basic cognitive tools for understanding LLMs”

Revision 2: we have added clarifications:

> “The choice of source domain provides the conceptual scaffolding, determining the questions that can be asked, the phenomena that are foregrounded, and the insights that can be adequately accessed.”

> “Notably, identifying these studies as metaphorical does not imply that the research conducted within them lacks formal rigor. The usage of “metaphor” in our analysis is anchored in the Lakoff and Johnson’s sense, serving as a meta-scientific conceptual scaffolding of each interdisciplinary inquiry.”

Reviewer's Comment #2

Second, the proposed concept of "machine experientialism" is presented as the main theoretical contribution, yet it remains somewhat underdeveloped. Although the author(s) frame it as a way to move beyond the dichotomy between “genuine understanding” and “pattern matching,” the framework is primarily articulated at a philosophical level. It remains unclear how adopting this perspective would concretely guide empirical research or generate new testable predictions about LLM behavior. The brief connection to Bayesian cognition and active inference is intriguing but not developed sufficiently to anchor the proposal within established cognitive science theory.

Response #2

We thank the reviewer for this constructive comment. We agree that our original articulation of machine experientialism leaned too heavily on philosophy and left its empirical utility underdeveloped. In the revised manuscript, we have addressed this limitation through two main revisions.

> “This philosophical stance finds a parallel in cognitive science, particularly the Bayesian cognition school [63]. A cognitive system is characterized as being separated from its environment by a boundary (Markov blanket) that mediates all interactions between the system's

internal states and the external world [64]. Without direct access to the environment beyond this boundary, it must construct and optimize an internal model that infers the hidden causes of the sensory signals it receives [65,66]. The internal model is jointly shaped by the structure of the external environment and the representational capacity of the system itself, which abstracts the experientialist insight into a substrate-independent principle.

Therefore, an extended experientialism can be articulated: cognition is the proactive subjective construction of the external world through the intelligent system's internal representational system. For humans, the sensorimotor system embedded in a physical and cultural environment gives rise to the embodied cognition that Lakoff and Johnson describe. For LLMs, the Transformer situated in the training corpus gives rise to a distinct mode of internal construction. This reading allows us to understand human and machine cognition in a comparative way; they can be analyzed through the same theoretical lens yet on independent terms."

Revision 4: to address how this machine experientialism could guide empirical research, we added the implication of our case study of temporal cognition, elaborating how machine experientialism guides a comparative research framework between human mind and LLMs.

""… This study instantiates the comparative methodology under the machine experientialism. The human-like temporal cognition is traced to specific overlaps between physical temporal sensory and human generated language, and between human brain's coding mechanisms and Transformer's information encoding. The study of machine cognition turns from a binary judgment (does LLM understand time?) into investigation of how different cognitive systems construct meaning of one concept under different constraints."

Revision 5: we also revised the text to articulate the predictive and practical implications of machine experientialism that "stochastic parrots" or "genuine understanding" cannot produce.

"…a medical AI might achieve high accuracy not through human's clinical logic but by exploiting another path with certain correlations [72]. LLMs undergoing standard safety alignment can exhibit behavioral compliance while maintain indifference in the latent representation [73]. As LLMs are ubiquitously deployed in consequential domains, the machine experientialism calls for a non-human centric cognitive science. This represents an inversion of research stance from cataloging human-like capabilities to understanding the machine's own internal construction, examining the convergence with humans for implementing everyday tasks reliably and divergence from humans for predicting potential risks. Only by grasping the unique logic of its internal reality, can we anticipate the cause of divergence, intervene and mitigate the risks, and guide it toward the beneficial ends."

Reviewer's Comment #3

Third, the manuscript's treatment of psychological research on LLMs appears somewhat narrow (and potentially outdated). The authors suggest that the psychology metaphor often leads to questionnaire-based approaches that attribute human-like traits such as personality or morality to LLMs. While such studies exist and may indeed raise concerns about anthropomorphic interpretation, this characterization overlooks a substantial and rapidly growing body of experimental work that applies established cognitive paradigms to LLMs. For example, recent research has used metamemory paradigms such as judgments of learning to compare human and LLM cognition and has demonstrated systematic differences in metacognitive monitoring between the two (Huff & Ulakci, 2025). Similarly, other studies use reasoning tasks, perception-like judgments, or decision paradigms drawn from cognitive psychology (Uhler et al., 2026). These approaches do not rely on questionnaire-style anthropomorphism but instead employ experimentally grounded behavioral methods. As a result, the manuscript may partially reflect an earlier stage of the literature and would benefit from acknowledging the broader experimental tradition emerging in "machine psychology".

Response #3

We thank the reviewer for highlighting the importance of experimentally grounded work in machine psychology. We would like to clarify that our manuscript devotes discussion to this experimental tradition. In the "The behavioral view from without" section, we distinguished two different paradigms in machine psychology: task- and questionnaire- based. We indicated that tasks, such as MCMC, similarity judgment task, two-step-reversal bandit task, and deliberation-harmful tasks, can chart both the convergence and divergence between human and machine cognition. And we used the contrast between task- and questionnaire- based approaches to illustrate where the psychology metaphor remains grounded in its minimal valid assumptions versus where it overextends into unverified domain.

We appreciate the reviewer's suggestion that this distinction could be made more prominent, and that additional examples would strengthen our characterization of the task-based paradigm.

Revision 6: in the revised manuscript, we added references to recent work such as judgments of learning, reasoning and decision tasks, and prototype judgment tasks:

> "⋯ judgments of learning study LLM's metacognitive monitoring [41]; decision-making tasks indicate an addition bias shared by human and LLMs [41]; prototype theory inspired study reveals distinct conceptual representations within LLMs [42]. By grounding comparison in measurable behaviors, these experimental methods can chart both the convergence and divergence between human and machine cognition."

Reviewer's Comment #4

Finally, the manuscript positions itself as a synthesis across disciplines, yet the literature coverage is somewhat imbalanced. Several references come from preprints or informal sources, while some relevant peer-reviewed work in cognitive science and psychology is not discussed. A more systematic engagement

with this literature would strengthen the conceptual argument and situate the proposed framework within ongoing debates about LLM cognition.

Response #4

We thank the reviewer for this suggestion. We acknowledge that our reference list includes several preprint and perspective from prominent researchers, and we appreciate the opportunity to clarify this choice. The field of LLM research has a distinctive publication ecology. A substantial proportion of high-impact, foundational work originates from industry labs such as Anthropic, Google DeepMind, and OpenAI. They are predominantly disseminated as preprints on arXiv and often remain unpublished in peer-reviewed venues yet constitute essential primary sources for serious discussion. We wish to assure that all cited preprints were carefully evaluated for methodological rigor before inclusion. We agree that a more thorough engagement with the peer-reviewed literature in cognitive science and psychology would strengthen our framework's grounding in established disciplinary debates. In the revised manuscript, we have supplemented our discussion with additional peer-reviewed references from these fields, as illustrated in Revision 6.

Reviewer's Comment #5

The balance between sections could be improved. The mechanistic perspectives receive relatively detailed discussion, whereas the behavioral and interactive perspectives are treated more briefly. Given that the central argument concerns the comparison of disciplinary lenses, a more balanced approach would strengthen the paper's structure.

Response #5

We thank the reviewer for this observation regarding the structural balance of the manuscript. We believe that the perceived imbalance stems primarily from a presentational issue. Our framework organizes the discussion across three analytical scales (mechanistic, behavioral, and interactive) each grounded in distinct disciplinary metaphors. The mechanistic scale encompasses two complementary disciplinary lenses (physics and neuroscience), whereas the behavioral and interactive scales each center on one primary lens (psychology and sociology, respectively). This results in a longer mechanistic section, which may give the impression of uneven treatment, even though the depth of discussion per discipline is comparable across the manuscript.

Revision 7: to address this concern, we have revised the structure of the mechanistic section by adding two sub-headings to make the two-discipline composition of this scale immediately visible to the reader:

> "**The mechanistic view from within**
>
> *Physics of LLM*

> This view adopts a macroscopic viewpoint, studying …
>
> *Neuroscience of LLM*
>
> Moving from a statistical whole to functional parts, *Neuroscience of LLM* presents …"

This reorganization clarifies that the greater length of the mechanistic section reflects its broader disciplinary scope. We believe this structural adjustment makes the parallel architecture of the framework more transparent and addresses the reviewer's concern .

Reviewer's Comment #6

Regarding open science practices, the manuscript does not address transparency, reproducibility, or accessibility in LLM research. Given the increasing reliance on proprietary models (e.g., OpenAI's ChatGPT) and opaque training pipelines, these issues are highly relevant to interpreting and evaluating claims about model cognition. A brief discussion of these challenges would strengthen the broader methodological perspective.

Response #6

We thank the reviewer for raising this important point. We agree that issues of transparency, reproducibility, and accessibility are increasingly central to LLM research, particularly given the growing dominance of proprietary models with opaque training pipelines.

The primary aim of our manuscript is to develop a conceptual framework for understanding how different disciplinary metaphors operate across analytical scales. The reviewer's suggestion intersects meaningfully with our cross-scale framework. The proprietary nature of many leading models constrains which analytical scales are available to researchers. For instance, when model weights and training data are inaccessible, the mechanistic scale (physics and neuroscience of LLM) becomes largely inapplicable, and researchers are effectively confined to behavioral or interactive scales. This asymmetry in analytical access is itself a consequence of the transparency challenges the reviewer identifies.

Revision 8: in the revised manuscript, we have added a brief discussion acknowledging this constraint:

> "Yet, these mechanistic viewpoints cannot fully grasp model's behavioral patterns in real-world application… Also, as frontier models become increasingly proprietary, mechanistic approaches may be constrained, channeling research toward behavioral and interactive scales, which risks shaping the types of LLM understanding of general researchers and public."

Dear Reviewer 1,

We sincerely thank you for your further involvement and thoughtful feedback. We have revised our manuscript correspondingly as below. **All changes are tracked using change mode in the revised manuscript.**

Reviewer's Comment

One small issue I still have trouble understanding concerns the claim that "machine experierentialism" commits one to "refusing the objectivist presupposition of mind-independent truth and the subjectivist assumption of unconstrained imaginative construction." The authors write at line 85, "This cognitive process advances the philosophy of experientialism, a middle path between objectivism and subjectivism, claiming that meaning, truth, and rationality are neither properties of the world nor arbitrary creations of our minds. Instead, they emerge from the interaction between our embodied selves and the world we live." This strikes me as a very bold claim, especially if it is meant to reject the existence of mind-independent truths or properties of the world. I wonder whether the authors really intend to endorse such a strong position. One can hold that machines interact with the world differently from the way humans do while still accepting that there are mind-independent facts about the world. My worry is that denying mind-independent facts may unnecessarily weaken the authors' argument.
There is also a familiar self-reference problem here. Is the authors' claim about machine experientialism itself meant to be a true claim about the actual world, perhaps corresponding to a scientific fact that holds independently of our perspectives? If so, then it seems to presuppose at least some mind-independent facts (e.g. the authors' claim). I therefore encourage the authors to clarify whether they mean to reject mind-independent truth altogether, or only to reject a particular objectivist conception of meaning.

Response

We sincerely thank the reviewer for the incisive observation that our original formulation might be unnecessarily strong. Indeed, we do not intend to deny the existence of "mind-independent truth" about the world itself. For our argument in this manuscript, a far more moderate claim would have been enough: the same external reality mediated through different representational systems gives rise to different internal constructions and understandings. We accept the external reality exists and constrains cognition.

Following the Reviewer's suggestion, we have accordingly weakened the relevant passages and deleted the expressions such as "mind-independent truth". The revised formulations now read:

"This cognitive process advances the philosophy of experientialism, a middle path between objectivism and subjectivism, claiming that understanding is not simply a given property of the world waiting to be discovered, nor merely an arbitrary creation of an unconstrained mind. Instead, it emerges from the interaction between the two. The same external reality mediated through different representational systems gives rise to different subjective understandings."

“It dissolves the long-standing debate between objectivism and subjectivism by (1) preserving objectivism's insistence that external reality constrains cognition and subjectivism's recognition that meaning is always meaning to a cognitive agent, while (2) refusing the objectivist presupposition that understanding is an unmediated reflection of the external world and the subjectivist assumption that it is an unconstrained imaginative construction. In this view, the specific mode of interaction with the environment shapes the representational systems through which we understand the world.”

We believe this revised position address the reviewer’s self-reference concern. It accepts that the external reality exists and constrains cognition. Machine experientialism aims to analyze how different cognitive systems construct their understanding of one external world through distinct representational architectures. We are grateful to the Reviewer for pressing this point, which helped us make the machine experientialism more restrained and precise.

Dear Reviewer 2,

We sincerely thank you for your further involvement and thoughtful feedback. We have revised our manuscript correspondingly as below. **All changes are tracked using change mode in the revised manuscript.**

Reviewer's Comment

Although the manuscript briefly acknowledges task-based approaches, their role is underemphasized relative to questionnaire-based studies. This creates an imbalanced picture of the field and risks giving readers the impression that psychological research on LLMs is primarily anthropomorphic in nature. Given that the manuscript positions itself as a cross-disciplinary synthesis, a more balanced treatment of experimental cognitive approaches is necessary.

This imbalance is especially evident in the paragraph discussing the use of LLMs as surrogates for human participants. The manuscript states that this "mistake is already spreading to psychology, sociology, and economics," raising concerns about reproducibility and reliability. While these concerns are important and well-motivated, the current formulation risks overstating the extent and nature of the problem. In particular, it does not sufficiently distinguish between uncritical uses of LLMs as human substitutes and more recent, theoretically grounded approaches that explicitly investigate where LLM behavior converges with or diverges from human cognition (e.g., Huff & Ulakci, 2025; https://www.nature.com/articles/s41598-025-22290-x). In cognitive psychology, LLMs are increasingly used not as surrogates in a strong sense, but as tools for probing cognitive processes under controlled conditions. These approaches directly address the concerns raised by the authors, rather than exemplifying the "mistake" described. Clarifying this distinction would make the argument more precise and avoid giving the impression that current research practices are overly generalized.

A substantial (and growing) body of work applies established experimental paradigms from cognitive psychology to LLMs, including similarity judgments, decision-making tasks, and metacognitive paradigms. These approaches do not rely on anthropomorphic assumptions but instead use behavioral methods to generate falsifiable comparisons between human and model cognition. For example, recent work using metamemory paradigms demonstrates systematic differences in metacognitive monitoring between humans and LLMs.

Addressing this point would substantially strengthen the manuscript's central argument by ensuring that the critique of "metaphor" is directed at specific methodological assumptions rather than at psychological approaches more broadly.

Response

We thank the reviewer for this comment. We agree that our original formulation “this mistake is already spreading to …” was overly broad and risked conflating the two distinct research practices in cognitive psychology. As the original manuscript in the Section “Deconstructing anthropomorphism with experientialism” states:

“At the same time, given the distinct construction process, representational system, and environment between human and LLM, to demand that LLM perfectly replicates human traits is a **category errors**. This **mistake** is already spreading to psychology, sociology, and economics, where LLM is increasingly used as AI surrogates for empirical studies [49,71,72], raising concerns about the reproducibility of such results in human participants and their overall reliability [73,74].”

Our original target of “”mistake” is the “category errors” that directly treats LLMs output as human data without empirical validation. The rigorous cognitive psychology research the reviewer recommends Huff & Ulakci (2025), which we have already cited as [41], is definitely not an instance of the category error we describe. This type of research empirically tests the cognitive equivalence between humans and LLMs. It therefore falls outside the scope of the category error we describe, and in fact aligns with the comparative methodology our framework advocates. The reviewer is right that distinguishing the two practices would make our argument more precise

Realizing this potentially inaccurate statement, we have revised the passage and removed the mentioning of specific disciplines to avoid this misunderstanding:

“… to demand that LLM perfectly replicates human traits is a category error. This error manifests in the uncritical use of LLMs as direct surrogates for human participants in empirical studies. Recent critiques cautioned that treating model outputs as interchangeable with human behavioral data without verifying the underlying cognitive equivalence [49,71,72] may compromise the reproducibility of findings in human populations and their overall reliability [73,74]. In contrast, the task-based cognitive approaches discussed earlier in this paper [36,38–42] do not commit this error. They use established experimental paradigms to empirically test whether human and LLM cognition converge or diverge on specific dimensions, without presupposing cognitive equivalence as a starting point. This distinction maps onto the minimal-assumption versus unverified-extension boundary delineated in Table 1.”

The original manuscript already devotes a full paragraph to task-based experimental approaches, listing similarity judgments, bandit tasks, metacognitive monitoring, decision-making tasks, and prototype-theory studies, and explicitly states that these methods ‘can chart both the convergence and divergence between human and machine cognition.’ The subsequent paragraph then draws the contrast with questionnaire-based approaches, and the third paragraph generalizes this into our minimal-assumption versus unverified-extension distinction.

With the revision to the ‘surrogates’ passage quoted above, which now explicitly endorses experimental cognitive approaches, such as those implemented in Huff & Ulakci (2025), we believe the manuscript provides a more balanced and consistent treatment of experimental cognitive research throughout. We thank the reviewer for this suggestion and recommendation, which strengthen the fairness and empirical grounding of our manuscript.
